# Varying efficacy of artesunate+amodiaquine and artesunate+sulphadoxine-pyrimethamine for the treatment of uncomplicated falciparum malaria in the Democratic Republic of Congo: a report of two in-vivo studies

**DOI:** 10.1186/1475-2875-8-192

**Published:** 2009-08-10

**Authors:** Maryline Bonnet, Ingrid van den Broek, Michel van Herp, Pedro Pablo Palma Urrutia, Chantal van Overmeir, Juliet Kyomuhendo, Célestin Nsibu Ndosimao, Elizabeth Ashley, Jean-Paul Guthmann

**Affiliations:** 1 Epicentre, Rue de Lausanne 78, CH-1211 Geneva 21, Switzerland; 2 Epicentre, 8 rue Saint Sabin, 70011, Paris, France; 3 Center for Infectious Disease Control, National Institute for Public Health and the Environment, PO Box 1, 3720 BA Bilthoven, the Netherlands; 4 Médecins Sans Frontières, Medical department, Brussels, Belgium; 5 Médecins Sans Frontières, Medical department, Barcelona, Spain; 6 Prince Leopold Institute of Tropical Medicine, Department of Parasitology, Antwerp, Belgium; 7 Mbarara University of Science and Technology, Mbarara, Uganda; 8 National Malaria Control Programme, Kinshasa, Democratic Republic of Congo; 9 Unité des Maladies à Prévention Vaccinale, Département des Maladies Infectieuses, Institut de Veille Sanitaire (INVS), 12, rue du Val d'Osne, 94415 Saint-Maurice cedex, France

## Abstract

**Background:**

Very few data on anti-malarial efficacy are available from the Democratic Republic of Congo (DRC). DRC changed its anti-malarial treatment policy to amodiaquine (AQ) and artesunate (AS) in 2005.

**Methods:**

The results of two in vivo efficacy studies, which tested AQ and sulphadoxine-pyrimethamine (SP) monotherapies and AS+SP and AS+AQ combinations in Boende (Equatorial province), and AS+SP, AS+AQ and SP in Kabalo (Katanga province), between 2003 and 2004 are presented. The methodology followed the WHO 2003 protocol for assessing the efficacy of anti-malarials in areas of high transmission.

**Results:**

Out of 394 included patients in Boende, the failure rates on day 28 after PCR-genotyping adjustment of AS+SP and AS+AQ were estimated as 24.6% [95% CI: 16.6–35.5] and 15.1% [95% CI: 8.6–25.7], respectively. For the monotherapies, failure rates were 35.9% [95% CI: 27.0–46.7] for SP and 18.3% [95% CI: 11.6–28.1] for AQ. Out of 207 patients enrolled in Kabalo, the failure rate on day 28 after PCR-genotyping adjustment was 0 [1-sided 95% CI: 5.8] for AS+SP and AS+AQ [1-sided 95% CI: 6.2]. It was 19.6% [95% CI: 11.4–32.7] for SP monotherapy.

**Conclusion:**

The finding of varying efficacy of the same combinations at two sites in one country highlights one difficulty of implementing a uniform national treatment policy in a large country. The poor efficacy of AS+AQ in Boende should alert the national programme to foci of resistance and emphasizes the need for systems for the prospective monitoring of treatment efficacy at sentinel sites in the country.

## Background

The Democratic Republic of Congo (DRC) is a vast country covering more than two million square kilometres and with an estimated population of 60 million people. In 2006, the malaria case fatality rate in children below five years of age was 0.61 [1]. Very few evaluations of the efficacy of artemisinin-based combinations have been conducted in DRC. In 2004, one study presented day 28 PCR genotyping-adjusted failure rates of 19.7% for artesunate+sulphadoxine-pyrimethamine (AS+SP) and 6.7% for AS+amodiaquine (AS+AQ) in South Kivu province [2]. Another study conducted in Rutshuru, a district near the Rwandan border in Eastern DRC, reported day 14 PCR- unadjusted cure rates of 63% and 24% for SP monotherapy and AS+SP, respectively [3].

The results of two *in vivo *efficacy studies conducted independently in DRC in 2003–2004, when the National Malaria Control Programme (NMCP) was in the process of changing treatment policy to an artemisinin combination therapy (ACT) are presented. The NMCP introduced AS+AQ as their first-line regimen in 2005 to replace SP monotherapy for the treatment of uncomplicated malaria. For one study, results on the molecular markers of SP resistance are also presented. A good correlation has been shown between mutations in the *dihydrofolate reductase *(*dhfr*) and *dihydropteroate synthase *(*dhps*) genes of *Plasmodium falciparum *and resistance to pyrimethamine and sulphadoxine, respectively [3-5].

## Methods

### Study site and population

**Figure 1:**
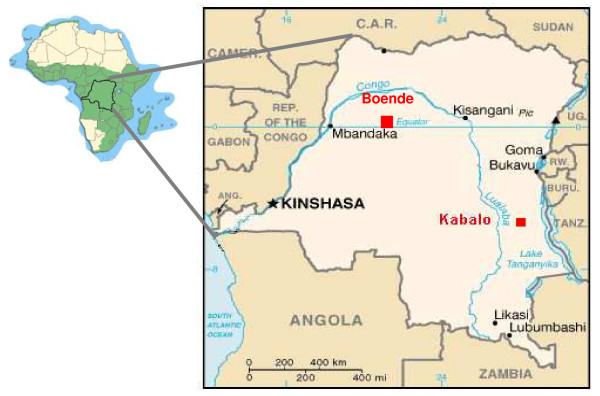
**Location of study sites**.

The first study was located in Boende, Equatorial province, in the northwest of the country and the second study in Kabalo, Katanga province, in the southeast of DRC (Figure 1). Malaria is endemic in DRC with perennial transmission in Boende and seasonal transmission in Kabalo, with a peak between November and May. The ethics committee of the NMCP of the Democratic Republic of Congo approved the two studies. The ethics review board of Médecins Sans Frontières also reviewed the study protocol for Kabalo. Written informed consent was obtained from children's carers before enrolment.

### Study design and patients' follow-up

The two studies were open-label. The study methodology was based on the WHO protocol for assessing efficacy of anti-malarials in areas of high transmission [6]. In Boende, SP and AQ monotherapies were evaluated as well as in combination with artesunate (AS+AQ and AS+SP). In Kabalo, SP monotherapy, AS+AQ and AS+SP were evaluated. There were slight methodological differences between the two studies.

#### Eligibility criteria

In Boende, eligibility criteria were age between six and 59 months, axillary temperature ≥ 37.5°C, living in or within 5 km of Boende city and *P. falciparum *parasitaemia between 2,000 and 200,000/μl. In Kabalo, due to the unstable security situation and recruitment difficulties, it was decided to extend the age group for inclusion to between six months and 10 years and to accept *P. falciparum *parasitaemia between 1,000 and 200,000/μl. Only children living less than half an hour's drive from the study clinic in Kabalo city were enrolled. In both studies, signs of severe malaria or any other serious health condition, intake of antimalarial treatment in the last seven days and mixed malaria infection were exclusion criteria.

#### Sample size and randomization

Sample size was calculated using Epi Info 6 software (Centres For Disease Control, Atlanta, USA). For the study in Boende, a sample size of 106 patients per arm was calculated using an estimated failure rate of 15%, a risk α of 5%, a precision of 7% and assuming 10% lost to follow-up. In Kabalo, it was 60 patients per arm, using 15% failure rate, 5% risk α, 10% precision and 15% lost to follow-up. In Boende, monotherapies and combination therapies were evaluated sequentially because of the concern that the study would be terminated early due to security concerns. Therefore, children were initially randomly allocated to SP or AQ, and then to AS+SP or AS+AQ, using a ratio of 1:1 each time. In Kabalo, patients were allocated randomly to AS+AQ, AS+SP or SP using a ratio of 1:1:1. In both studies, randomization was in blocks of 12 and treatment allocations were concealed in sealed opaque envelopes, opened after consent was obtained.

#### Treatment

After randomization, haemoglobin, asexual parasitaemia and presence of gametocytes were recorded at day 0, and patients received the first dose of treatment: (i) SP (Fansidar^®^, Roche, Basel, Switzerland) was given as a single dose of approximately 1.25 mg/kg of pyrimethamine and 25 mg/kg of sulphadoxine; (ii) AQ (Camoquin^®^, Pfizer, Dakar, Senegal) was given at a dose of 10 mg/kg/day once daily for three days; and (iii) AS (Arsumax^®^, Sanofi Winthrop AMO, Gentilly, France) was given at a dose of 4 mg/kg once daily for three days. All doses were directly observed at the health centre. Patients who vomited within half an hour had the full dose replaced; if vomiting occurred between 30 minutes and one hour after intake, only half the dose was replaced. Study drugs manufactured according to Good Manufacturing Practice standards were purchased for the purpose of the studies. A capillary blood sample was also collected onto Whatman grade 3 filter paper and stored on site for genotypic analysis if needed.

#### Participants' follow-up

Children were re-assessed clinically on days 1, 2, 3, 7, 14, 21, 28 and parasitologically on days 2, 3, 7, 14, 21 and 28 after the start of treatment. Participants were encouraged to access the study clinic at any time between scheduled visits in case of fever or any health problem. Indications for withdrawal from the study were (i) vomiting any dose of study drug twice (at which point intravenous quinine was given), (ii) serious allergic reaction to the study drug, (iii) other serious illness, (iv) intake of any drug with anti-malarial properties during the period of follow-up, (v) withdrawal of consent or (vi) failure to attend scheduled visits. Treatment failures were treated with quinine hydrochloride. The WHO classification of treatment outcomes was used with patients categorised as early treatment failures (ETF), late clinical failures (LCF), late parasitological failures (LPF) on day 28 or adequate clinical and parasitological response (ACPR) [6]. In the event of symptomatic parasite recurrence (LCF) between days 10–28 of follow-up or LPF, a second blood sample for polymerase chain reaction (PCR) genotyping was collected (recurrences on or before day 9 were assumed to be recrudescences). The genotype of malaria parasites collected at day 0 and at the time of parasitaemia recurrence was compared in order to investigate if the recurrence resulted from recrudescence or reinfection.

### Laboratory procedures

In Boende, thick blood films were stained with Giemsa 10% for 10 to 20 minutes and with Giemsa 5% for 25 minutes in Kabalo. Parasite density was determined as the number of parasites per 200 white blood cells (WBC), assuming a total WBC count of 8,000/μl. If less than 10 parasites were read per 200 WBC, the count was extended to 500 WBC. Microscopists reading the slides were blind to the treatment allocation. In the two studies, there was an independent double reading of all slides and resolution of discordant results (negative/positive/species or parasitaemia discordance above 50%) by a third senior reader. The third reading was taken as the final result. In addition, an external quality control of 10% of slides selected randomly from the screening, inclusion and follow-up slides was performed. From the study in Boende, slides were sent to the Institute of Tropical Medicine (IMT), Antwerp (Belgium). Slides from Kabalo were sent to the Amikuvu Regional Public Health Laboratory of Goma and the National Institute of Biomedical Research of Kinshasa (DRC). Haemoglobin was measured using the Lovibond technique (Assistant Co., Sondheim Rhon, Germany).

PCR genotyping to distinguish recrudescence from reinfection was performed in two different laboratories. For Boende, the analysis was performed at the ITM of Antwerp, Belgium using merozoite surface protein-1 (*msp1*) and merozoite surface protein-2 (*msp2*) *P. falciparum *gene loci [7]. Infections were defined as recrudescent if at least one common band for both markers was observed in the presenting and recurrent infections. Recurrences were defined as reinfection if completely different bands were observed between the two infections in at least one of the two markers. For the study in Kabalo, genotyping was performed at the Epicentre laboratory at Mbarara University, Uganda according to a published method using three *P. falciparum *gene loci: *msp1*, *msp2*, and glutamate rich protein (*glurp*) [8]. The *glurp *alleles were first compared between baseline and post-treatment samples. If they were different, the recurrence was attributed to a reinfection. If they were identical, then the *msp1 *and *msp2 *alleles were compared between the 2 samples. If there was a common band on one or two *msp *markers, then the recurrence was considered as recrudescence. If not, it was due to a reinfection.

In Boende, additional blood samples were collected from the children treated with SP as part of a multi-centre genotyping study to look for molecular markers of antifolate resistance. Analysis of mutations in codons 51, 59 and 108 of the dihydrofolate reductase (*dhfr*) and in codons 437 and 540 of the dihydropteroate synthase (*dhps*) genes of *P. falciparum *was performed in the ITM of Antwerp, Belgium using a real mutation-specific nested PCR and/or restriction digestions [9,10]. The classification of samples was based on a published methodology [11]. Each *dhfr *and *dhps *codon was characterized as 'wild type' (no mutation present), 'mixed' (both wild and mutant genotypes clearly present in the same infection), or 'pure mutant' (only mutant genotypes detected). The *dhfr *genotypes for each infection were categorized as follows: 'wild type': no mutation detected; 'single': infection involving parasites with a single mutation; 'double': infection involving parasites with a double mutations and 'triple': infection involving parasites with all three mutations detected. The *dhps *genotypes were defined as wild type; single or double mutations. Infections by parasites with a triple *dhfr *mutation and double *dhps *mutation were categorized as 'quintuple' mutation infections. These results have been reported in detail elsewhere [12,13].

### Analysis

Data were entered in Microsoft Excel^® ^and analysed with Stata^® ^10 (College Station, Texas, USA). Failure rates were estimated using Kaplan-Meier survival analysis, in which withdrawals, losses to follow-up and reinfections were censored on the last day seen. Patients with missing or indeterminate PCR results were censored on the closest prior visit when the malaria smear result was negative.

## Results

### Boende study

**Figure 2:**
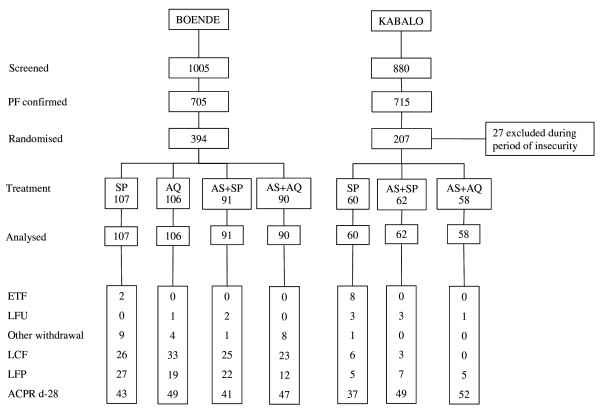
**Details of inclusions and follow-up in thestudies in Boende and Kabalo**.

A total of 1,005 children were screened between September 2003 and February 2004, of whom 394 (39.2%) were enrolled (Figure 2). Three patients (0.8%) were lost to follow up. Baseline characteristics are presented in Table 1. Trends in fever, parasitaemia and haemoglobin over time are presented in Table 2. More than one quarter of patients treated with AS+SP or AS+AQ were parasitaemic on day 7. Of 68 children still parasitaemic on day 7, 3 had a fever and were classified as failures. The day 28 unadjusted failure rates were 55.5% [95% confidence interval CI: 46.0–65.4] for SP, 25.7% [95% CI: 18.3–35.4] for AQ, 52.2% [95% CI: 42.3–62.9] for AS+SP and 41.0% [95% CI: 31.3–52.3] for AS+AQ. PCR genotyping results are shown in Table 3. The end-point classifications after PCR adjustment are shown in Table 4. For the monotherapies, PCR adjusted failure rates were 35.9% [95% CI: 27.0–46.7] for SP and 18.3% [95% CI: 11.6–28.1] for AQ. Failure rates on day 28 after PCR-genotyping adjustment of AS+SP and AS+AQ were estimated as 24.6% [95%CI 16.6–35.5] and 15.1% [95%CI: 8.6–25.7], respectively. There were 2 (2.6%) ETFs in the SP arm and none in the other arms. Approximately half of all patients treated with SP, AQ or AS+SP required re-treatment by day 28. No child developed severe malaria after enrolment and there were no deaths. Three children were withdrawn from the study in the first week due to serious concomitant illness- pneumonia, dysentery and suspected meningitis.

**Table 1:** Baseline characteristics of included patients in the studies in Boende and Kabalo

	Boende				Kabalo		
Characteristics N	SP 107	AQ 106	AS+SP 91	AS+AQ 90	SP 60	AS+SP 62	AS+AQ 58
Males, n (%)	56 (52.3)	54 (50.9)	45 (49.5)	46 (51.1)	26 (43.3)	37 (59.7)	36 (62.1)
Age months median [range]	18 [6–59]	19 [6–59]	20 [6–56]	21 [6–56]	36 [8–96]	25 [7–108]	33.5 [7–108]
Weight kg median [range]	9.2 [5.1–18]	10.3 [5.8–22]	9.7 [5.9–17]	9.9 [6.1–16.5]	11 [6–25]	11 [6.7–22]	11 [5.7–26]
MUAC mm median [range]	140 [112–172]	142 [110–176]	142 [118–170]	138 [124–170]	146 [116–194]	148 [122–180]	149 [116–180]
Asexual parasitaemia (/μl), GMR* [range]	26724 [2080–187200]	28992 [2097–189037]	26662 [2171–163840]	26134 [2073–186400]	22501 [1995–181970]	20352 [2455–162181]	19934 [2455–147911]
Temperature °C median [range]	38.5 [37.5–40.2]	38.6 [37.5–40.5]	38.5 [37.5–40.6]	38.7 [37.5–40.6]	38.4 [37.5–40.5]	38.4 [37.5–41.0]	38.4 [37.5–40.5]

*GMR = geometric mean

**Table 2:** Variations of fever, parasitaemia and haemoglobin over the time in the studies in Boende and Kabal

	Boende					Kabalo			
Characteristics N	SP 107	AQ 106	AS+SP 91	AS+AQ 90	*p*	SP 60	AS+SP 62	AS+AQ 58	*p*
Proportion febrile d1 (%)	20/106 (18.7)	9/105 (8.5)	8/91 (8.8)	5/89 (5.6)	0.01	9/59 (15.3)	5/62 (8.1)	5/58 (8.6)	0.37
Proportion febrile d2 (%)	21/103 (20.4)	3/105 (2.8)	3/91 (3.3)	2/87 (2.2)	<0.001	13/58 (22.4)	1/62 (1.6)	1/58 (1.7)	<0.001
Proportion parasitaemic d2(%)	84/103 (81.5)	90/105 (85.7)	60/91 (65.9)	56/87 (64.4)	<0.001	35/58 (60.3)	8/62 (12.9)	9/58 (15.5)	<0.001
Proportion parasitaemic d7(%)	12/97 (11.4)	6/98 (6.1)	25/89 (28.1)	25/85 (29.4)	<0.001	5/51 (9.8)	7/60 (11.7)	1/54 (1.8)	0.12
Hb d0 median [range]	9.7 [6.0–12.7]	9.7 [5.0–11.7]	9.7 [6.7–12.7]	9.7 [6.7–12.7]		10.7 [6.7–12.7]	10.7 [6.7–14.0]	10.7 [6.0–14.0]	
Anaemia* d0 (%)	100 (93.4)	102 (96.2)	83 (91.2)	83 (92.2)	0.51	31 (51.7)	39 (62.9)	36 (62.1)	0.38
Hb d28 median [range]	10.7 [6.7–12.7]	10.7 [6.7–12.7]	10.7 [7.0–12.7]	10.7 [7.3–12.7]		11.7 [10.7–12.7]	11.7 [9.7–12.7]	11.7 [9.7–14.0]	

* Defined by haemoglobin below 11 g/dL

**Table 3:** PCR genotyping results after day 9 in the studies in Boende and Kabalo

	Boende				Kabalo		
	SP	AQ	AS+SP	AS+AQ	SP	AS+SP	AS+AQ
Recurrences,(N)	53	51	47	35	11	10	5
Recrudescence, N(%)	31(58.5)	16(31.4)	20(42.5)	11(31.4)	3(27.3)	0	0
Reinfection, N(%)	19(35.8)	33(64.7)	20(42.5)	18(51.4)	5(45.4)	8(80)	4(80)
No PCR result*, N(%)	3(5.7)	2(3.9)	7(14.9)	6(17.1)	3(27.3)	2(20)	1(20)

*missing, indeterminate or failure of DNA amplification

**Table 4:** Failure rates at Day 28 after PCR adjustment in the studies in Boende and Kabalo

	Boende				Kabalo		
Outcome N	SP 76	AQ 66	AS+SP 61	AS+AQ 58	SP 48	AS+SP 49	AS+AQ 52
ETF, n (%)	2 (2.6)	0	0	0	8 (16.7)	0	0
LCF, n (%)	16 (21.0)	10 (15.1)	13 (21.3)	8 (13.8)	3 (6.3)	0	0
LPF, n (%)	1 (19.7)	7 (10.6)	7 (11.5)	3 (5.2)	0	0	0
Overall failure rate^1^ % [95% CI]	35.9 [27.0–46.7]	18.3 [11.6–28.1]	24.6 [16.6–35.5]	15.1 [8.6–25.7]	19.6 [11.4–32.7]	0 [0–5.8]^2^	0 [0–6.2]^2^

WHO 2001 outcome definitions used.

ETF: early treatment failure; LFU: lost to follow-up; LCF: late clinical failure; LFP: late parasitological failure; ACPRA: adequate clinical and parasitological response

^1 ^Estimated by Kaplan-Meier Survival Analysis, reinfections censored on day endpoint classified. Patients with missing or unresolved PCR results censored on last day seen with negative malaria smear result.

^2 ^One sided CI for a proportion calculated (Wilson method)

Out of a total of 179 slides sent for external quality control, 41 (22.9%) had positive-negative discordant results during follow-up, 27 (65.7%) of them with very low parasitaemia (<100/μL). In three cases (1.7%), the discordance affected classification of the patient's outcome: patients with adequate therapeutic response were reclassified as ETF (two cases) and LPF (one case) based on the results of the external quality control.

The results of the molecular markers of antifolate resistance are presented in Table 5. There was 3/102, 2.9% [95%CI: 0.6–8.3] quintuple *dhps *and *dhfr *mutation, one with pure (1%) and two with mixed mutations, respectively.

**Table 5:** Prevalence of mutations at codons 108, 51, 59 in *dhfr*, and of mutations at codons 437, 540 of *dhps *in the study in Boende, N = 102

	N	%	95% CI
*Dhfr*			
- Sensitive	3	2.9	0.6–8.3
- Single-mutant 108	2	2.0	0.2–6.9
- Doublemutant 108, 51	31	30.4	21.7–40.3
- Double-mutant 108, 59	4	3.9	1.1–9.7
- Triple mutant 108, 51, 59	62	60.8	50.6–70.3
○ Mixte	44	43.1	33.4–53.3
○ Pure	18	17.6	10.8–26.4
*Dhps*			
- Sensitive	30	29.4	20.8–39.2
- Single	68	66.7	56.6–75.7
- Double mutation 437, 540	4	3.9	1.1–9.7
○ Mixte	1	1.0	0.0–5.3
○ Pure	3	2.9	0.6–8.3

### Kabalo study

A total of 880 children were screened between June and December 2004, of whom 207 (23.5%) were enrolled (Figure 2). During the study, investigators had to be evacuated from the site between October and December 2004 due to political instability. Twenty-seven patients recruited during this time were excluded from the analysis because of non-adherence to the study protocol. Baseline characteristics were similar between the groups (Table 1). Variations of fever, parasitaemia and haemoglobin over time are presented in Table 2. About 10% of patients in the AS+SP arm were parasitaemic on day 7. None had fever. The day 28 unadjusted failure rates were 33.2% [95% CI: 22.6–41.1] for SP, 16.6% [95% CI: 9.3–28.7] for AS+SP and 8.8% [95% CI: 3.8–19.8] for AS+AQ. PCR genotyping results are shown in Table 3. The final end-point classifications after PCR adjustment are shown in Table 4. The PCR adjusted failure rate for SP monotherapy on day 28 was 19.6% [95% CI: 11.4–32.7]. Corresponding failure rates were 0 [1-sided 95% CI: 5.8] for AS+SP and AS+AQ [1-sided 95% CI: 6.2]. There were eight (16.7%) ETFs in the SP monotherapy arm and none in the other treatment arms.

No child developed severe malaria after enrolment and there were no deaths. There was one serious adverse event – a child diagnosed with acute severe malnutrition during the first week. Of 182 slides sent for external quality control, 26 (14.3%) had positive-negative discordant results during follow-up; none of them affected patients' outcomes.

## Discussion

These two independent studies document anti-malarial efficacy in 2004 from locations in the east and west of DRC, a country from which few such data have been reported previously. Efficacy of SP was poor for both sites. Efficacy of the AS-SP and AS-AQ combinations was poor in Boende but high in Kabalo. The comparison between the results of the two studies is limited by the difference in study methodology. The age and parasitaemia enrolment criteria were different and different genotypic methods were used to discriminate recrudescence from reinfection. Indeed, it is possible that the chance of being classified as a recrudescence was higher in Boende where a two-loci genotypic method was used compared to Kabalo, where a three-loci method was used. The high failure rate of SP in Boende is consistent with the molecular results for antifolate resistance, showing 61% had the triple *dhfr *mutation, which is known to be associated with an increased risk of treatment failure [3,4].

The threshold of resistance to SP at which it is no longer useful to add artesunate is not well defined. In Kabalo, the day 28-failure rate of SP was close to 20%, dropping to zero when given with three days of AS. Comparable results have been found in studies from Bie province in Angola where the PCR-adjusted 28-day failure rate was reduced from 25.3% for SP alone to 1.2% after addition of AS and in Southern Benin where the PCR-corrected failure rate of AS+SP at 28 days was reduced from 44.1% to 5.3% when combining AS to SP [14,15]. In contrast, in Boende the failure rate of SP alone was 36% and only dropped to 25% when AS was added.

It is not that surprising that in a large country, such as DRC, considerable differences in anti-malarial efficacy between provinces were observed. Site-specific patterns of treatment-seeking behaviour or malaria transmission intensity may explain geographical differences in the efficacy of anti-malarial treatments [16-18]. The higher reinfection rate and higher proportion of anaemic children in Boende suggest higher transmission intensity there.

A limitation of these studies is the disappointing external quality control results, which suggest a failure of the internal quality control system in place. In the case of the Boende study, it is important to note that for the majority of discordant results the parasitaemia detected by one of the laboratories was less than 100/μL. Also, these results affected the classification of outcome in only three cases in Boende and none in Kabalo. The delay of some months before the EQC will have resulted in a deterioration in slide quality, which may partially explain these poor results. Having an adequate standard of microscopy even in these circumstances is critical. The experience at these two sites suggests that internal quality control measures in place were not adequate to guarantee this standard. External quality control at regular intervals may be the answer but is expensive, labour-intensive and the second reading needs to be timely to avoid excessive deterioration of slides, or slides need to be mounted. Sending a random sample of slides seems an obvious approach but leads to problems when agreement is not high. It is then recommended that all the slides be sent, by which time several months may have elapsed since the study has finished.

Conducting *in vivo *efficacy studies in remote regions, in which there is political instability, is a major challenge. Proxy markers for resistance, such as molecular markers are invaluable tools to assess anti-malarial resistance in such a context, being less resource demanding than an *in vivo *study. Unfortunately, there are no markers, which reliably predict clinical resistance to drugs other than SP.

Data from neighbouring Congo-Brazzaville to the west indicate high level SP and AQ resistance [19,20]. Efficacy of AS+AQ in studies in Burundi and Tanzania to the East in 2002 and 2004 respectively was high, although in the Tanzanian study efficacy of amodiaquine alone was evaluated but not reported [21,22]. The observation of varying efficacy of the same anti-malarial in two different regions in the same country shows the problem of implementing a uniform national policy, especially in large country like DRC. While these results cannot be generalized to the whole provinces (Katanga or Equatorial) and even less to the whole country, they should alert the national programme to foci of resistance to AS+AQ, which became the national treatment policy in 2005. Efficacy of ACTs including partner drugs to which there is pre-existing resistance such as SP or AQ, need to be monitored at very regular intervals. According to the 2006 WHO Africa Malaria Report, eight sentinel sites are now operational in DRC [23].

Since these studies were performed WHO pre-qualified anti-malarials have become available, e.g. artemether-lumefantrine and AS+AQ fixed-dose combination (FDC). Dihydroartemisinin-piperaquine and the FDC of artesunate-mefloquine are in the process of being registered. Both artemether-lumefantrine and dihydroartemisinin-piperaquine have been evaluated in a number of African countries and appear highly efficacious [24,25]. The NMCP of DRC may need to consider regional adjustments to its policy guidelines, although such an approach is challenging. DRC is participating in the World Bank Booster programme worth $US30 million over four years, which finances provision of long-lasting insecticidal nets (LLINs), intermittent preventive treatment (IPT) for pregnant women and ACT as first-line treatment of malaria . It is important that concomitant monitoring of anti-malarial efficacy is supported in order to inform policy makers [26].

## Competing interests

The authors declare that they have no competing interests.

## Authors' contributions

MB was the principal investigator of the study conducted in Boende. She wrote the study protocol, coordinate supervised the study and wrote the paper. IVDB was the principal investigator of the Kabalo site. She wrote the study protocol, coordinate supervised the study and revised the manuscript. MVH participated to the development of the study protocol for Boende, gave a technical support to the investigators for the Boende site and revised the manuscript. PPPU participated to the development of the study protocol for Kabalo, gave a technical support to the investigators for the Kabalo site and revised the manuscript. CVO performed the genotypic analysis and coordinated the external quality control of the reading of malaria slides for the Boende site. She revised the manuscript. JK performed the genotypic analysis for the Kabalo site and revised the manuscript. CNN was the director of the National Malaria Control Programme when the two studies were discussed. He participated to the development of the study protocols and revised the manuscript. EA revised the statistical analysis of the 2 studies and revised the manuscript. JPG gave technical support to the principal investigators during the protocol writing, analysis and writing of the manuscript.

All authors have read and approved the final manuscript.
